# Murine Typhus Presenting with Acute Psychosis and Disseminated Intravascular Coagulation: A Case Report

**DOI:** 10.7759/cureus.4450

**Published:** 2019-04-13

**Authors:** Abdullah M Pervaiz, Rabail Tariq, Salman A Bangash, Yasir Lal

**Affiliations:** 1 Internal Medicine, University of Massachusetts Medical School - Baystate Medical Center, Springfield, USA; 2 Pediatrics, Beaumont Hospital, Royal Oak, USA; 3 Internal Medicine, Doctors Hospital / University of Texas Rio Grande Valley, Edinburg, USA; 4 Internal Medicine, Baylor St. Luke's Medical Center, Houston, USA

**Keywords:** rickettsial infection, murine typhus, dic, psychosis, hallucinations, rickettsia typhi, doxycycline, disseminated intravascular coagulation

## Abstract

Murine typhus is an endemic infectious disease caused by Rickettsia typhi and is transmitted by fleas. It typically causes a mild illness with symptoms of fever, rash, headache, chills, and non-specific gastrointestinal complaints. However, there have been no reported cases in the literature of murine typhus infection causing symptoms of acute psychosis and disseminated intravascular coagulation (DIC).

A 30-year-old female with a history of gastric bypass and chronic pain syndrome presented to the emergency department with altered mental state and fever. She developed vivid visual hallucinations, DIC, and hypoxia with pulmonary opacities, ultimately requiring intubation. Magnetic resonance imaging (MRI) showed leptomeningeal enhancement with unremarkable cerebrospinal fluid (CSF) studies. Serum murine typhus serology came back positive. Doxycycline therapy was initiated, which resulted in complete patient recovery.

This case shows that murine typhus infection may present with acute psychosis and can mimic DIC, leading to diagnostic confusion. MRI sequences may show leptomeningeal enhancement, which has never been reported before in patients with typhus. Early neurological imaging using advanced MRI sequences for patients presenting with altered sensorium, visual hallucinations, and symptoms similar to thrombotic thrombocytopenic purpura (TTP) may help with early diagnosis, decreased hospital stay, and better prognosis.

## Introduction

Murine typhus is a febrile illness caused by Rickettsia typhi. Rickettsia species are gram-negative, obligate intracellular bacilli. Murine typhus is uncommon in the United States but is seen at times in the southern parts, particularly California and Texas. Most cases occur during the summer or fall season [1]. The reservoir is found in rat fleas or feces; other animal sources include cats, opossums, skunks, and raccoons [1]. The vectors include human body lice, flying squirrel ectoparasites, ticks, and fleas [2].

Typhus usually manifests as fever (98%-100% patients), rash (50% patients), or a triad of fever, headache, and rash (12.5% patients) [3]. Other symptoms, involving multiple organ systems, are exceedingly rare.

Disease severity has been related to increased age, renal dysfunction, leukocytosis, hypoalbuminemia, and prior sulfa antibiotics use [3]. Common lab findings include azotemia/proteinuria, leukopenia (early stages), thrombocytopenia, mild hepatic transaminase elevations, hypoalbuminemia, and hyponatremia [4]. Definite diagnosis is done with polymerase chain reaction (PCR) testing.

Although murine typhus can present with acute delirium and encephalitis, there have been no reported cases of murine typhus presenting with acute encephalopathy, psychosis, leptomeningeal enhancement, vivid visual hallucinations, and disseminated intravascular coagulation. However, there has been one reported case of Scrub typhus presenting with visual hallucinations in India in 2015. There was another study conducted in the 1950s, which reported psychiatric abnormalities in typhus patients, including hallucinations, but none mentioned meningeal disease. We present a case of a patient with murine typhus presenting with fever, altered mental status, thrombocytopenia, and vivid hallucinations. All the presenting complaints subsided after treatment with doxycycline.

## Case presentation

**Figure 1:**
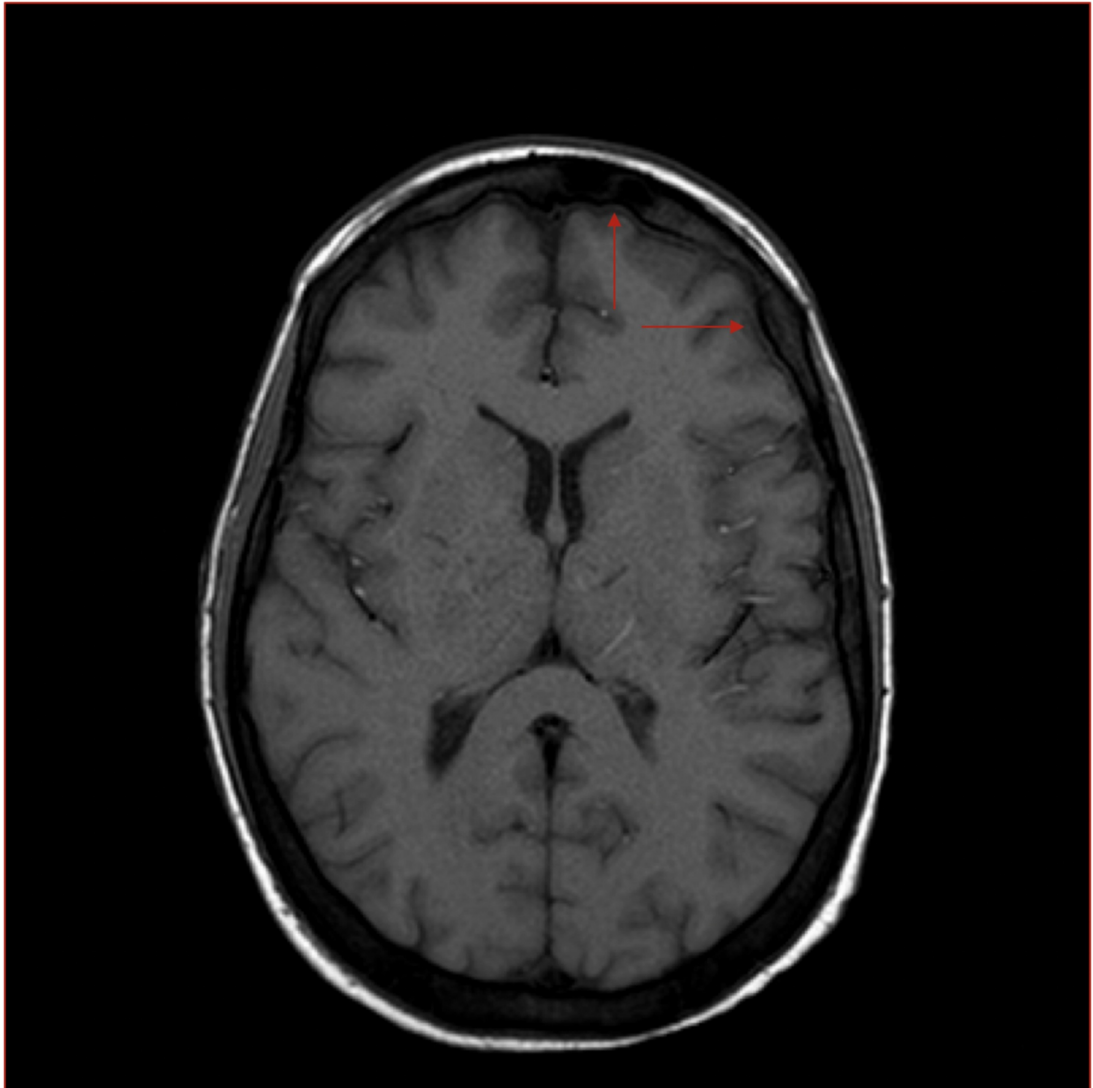
MRI of the brain revealing diffuse leptomeningeal enhancement consistent with meningitis or inflammatory changes

A 30-year-old female with a past medical history of gastric bypass and chronic pain syndrome presented to the emergency department with mental confusion and fever. Initial lab results showed thrombocytopenia with a platelet count of 80,000 and anemia with hemoglobin of 4.2 g/dl. A preliminary diagnosis of TTP was made, and the patient was admitted to the hospital for further management. Peripheral smear did not show any schistocytes. The patient subsequently developed worsening vivid visual hallucinations. Cefepime and vancomycin were empirically started for meningitis but the patient did not improve. Lumbar puncture was within normal limits. The patient’s condition worsened, and she became hypotensive with the development of DIC. Hypoxic respiratory failure ensued and the patient was intubated. Chest X-ray showed diffuse pulmonary opacities and MRI was positive for leptomeningeal enhancement consistent with meningitis or inflammatory changes (Figure 1). Typhus serologies came back positive and doxycycline was initiated, which led to rapid and complete resolution of symptoms, and the patient recovered.

## Discussion

Typical endemic typhus presents as an abrupt onset of fever, headache, and rash. Patients usually have a history of a flea bite or exposure to a natural disaster or war. Untreated typhus usually lasts about two weeks. Several months may pass before complete recovery from fatigue and malaise. Neurological symptoms are limited to mental dullness ranging to coma. Physical findings include fever, relative bradycardia, rash, or generalized lymphadenopathy. Common lab findings include azotemia/proteinuria, leukopenia (early stages), thrombocytopenia, mild hepatic transaminase elevations, hypoalbuminemia, and hyponatremia [4]. A definite diagnosis is done with PCR testing.

It`s not rare to find an atypical presentation of typhus. Our patient presented with a picture that mimics TTP. It’s very important for the physician to be vigilant of patients presenting with thrombocytopenia, which can be misdiagnosed as TTP. Although central nervous system (CNS) involvement is common in typhus, only one case report of typhus encephalomyelitis diagnosed on brain MR imaging has been published in the English language literature to our knowledge. In this case, the T2-weighted images depicted areas of signal hyperintensity in the dorsolateral pontomedullary region, bilaterally in the cerebellar peduncles, and in the cervical spinal cord [5]. Our patient, however, presented with leptomeningeal enhancement on MRI scan. Therefore, it is pertinent to note that a patient with endemic typhus may show leptomeningeal enhancement on MRI that may be confused for an inflammatory process such as meningitis.

In this case, we’d like to emphasize the role of early neurological imaging using advanced MRI sequences for patients presenting with altered sensorium, visual hallucinations, and symptoms similar to TTP. MRI sequences may show leptomeningeal enhancement, which has not presented before in patients with typhus. At this time, more research is required to prove that advanced neuroimaging techniques play a crucial role in this rare and life-threatening, yet treatable, disease.

## Conclusions

In patients with murine typhus presenting with altered sensorium, neurological complications should be suspected, as early diagnosis and treatment will alter the prognosis and reduce the mortality rate. Patients may present with vivid hallucinations and signs of aseptic meningitis. Thus, physicians should be vigilant about this atypical presentation. Advanced MRI sequences and clinical presentation play a pertinent role in diagnosing and treating the complications of this rare disease.
